# Comorbidity Factors and Brain Mechanisms Linking Chronic Stress and Systemic Illness

**DOI:** 10.1155/2016/5460732

**Published:** 2016-02-08

**Authors:** Vanja Duric, Sarah Clayton, Mai Lan Leong, Li-Lian Yuan

**Affiliations:** Department of Physiology and Pharmacology, Des Moines University, Des Moines, IA 50312, USA

## Abstract

Neuropsychiatric symptoms and mental illness are commonly present in patients with chronic systemic diseases. Mood disorders, such as depression, are present in up to 50% of these patients, resulting in impaired physical recovery and more intricate treatment regimen. Stress associated with both physical and emotional aspects of systemic illness is thought to elicit detrimental effects to initiate comorbid mental disorders. However, clinical reports also indicate that the relationship between systemic and psychiatric illnesses is bidirectional, further increasing the complexity of the underlying pathophysiological processes. In this review, we discuss the recent evidence linking chronic stress and systemic illness, such as activation of the immune response system and release of common proinflammatory mediators. Altogether, discovery of new targets is needed for development of better treatments for stress-related psychiatric illnesses as well as improvement of mental health aspects of different systemic diseases.

## 1. Introduction

Stress can be defined as the presence of acute or persistent physiological or psychological threats to the organism that results in significant strain on the body's compensatory systems. Goldstein and McEwen described stress as a condition where a discrepancy exists between the current or anticipated perceptions and expected perceptions of the internal or external environment [1]. Stress is further characterized by the existence of compensatory responses that generally deviate from and extend normal physiological regulation in order to protect the living organism against severe threats and sustain life.

To date, activation of the hypothalamic-pituitary-adrenal (HPA) axis has been widely accepted as one of the central physiological mechanisms involved in stress response. It is primarily dependent on stimulation of corticotrophin-releasing hormone (CRH) and adrenocorticotropic hormone (ACTH) release from the hypothalamic paraventricular nucleus (PVN) and the anterior pituitary, respectively, leading to increased production and systemic release of glucocorticoids from adrenal glands. However, high glucocorticoid levels that are associated with repeated or chronic stress can also lead to hyperactivation of the HPA axis due to diminished function of negative feedback mechanisms, especially within the limbic brain areas that regulate mood and emotional responses. Moreover, chronic stress is also accompanied by increased secretion of proinflammatory cytokines (PICs) that can further impair neuronal transmission and plasticity within these brain circuits;* for a more comprehensive review see* [2].

In contrast to coordinated physiological processes that underlie maintenance of steady state or homeostasis, prolonged psychological or traumatic stress can lead to disruption of cellular and systemic equilibriums resulting in dysfunction of both the nervous system and peripheral organ systems. Thus, chronic stress can ultimately lead to alterations and dysfunction of internal systems that control stress responses and consequent development of both neurological and psychosomatic illnesses. This concept is further supported by clinical reports indicating that psychological stress and systemic disorders are both commonly associated with adverse impact on mental health and development of comorbid psychiatric illnesses. Indeed, clinical depression is present in up to 50% of patients with chronic systemic conditions (e.g., pain, stroke, cardiovascular disease, obesity, diabetes, and cancer), much higher than the rate of 5–8% in the general population [3–5]. However, despite the abundance of empirical evidence and the well-established link between different physical disease states and mental illness, the question remains why so many different systemic disorders are frequently accompanied by deterioration in mental health. Adaptation to stress or allostasis, a well-known phenomenon that requires integration of autonomic, endocrine, and behavioral response systems, most likely plays an important role in such comorbidities; however, the precise underlying physiological and biochemical mechanisms are still poorly understood. Thus, in this review we will discuss the pathophysiological consequences of chronic stress on the brain and central nervous system (CNS) with particular focus on alterations in neural plasticity and function, as well as the correlation between different systemic illnesses and mental disorders such as clinical depression.

## 2. Effects of Chronic Stress on Neural Mechanisms Involved in Development of Mood Disorders

### 2.1. Changes in Neuronal Structure and Function

**Figure 1:**
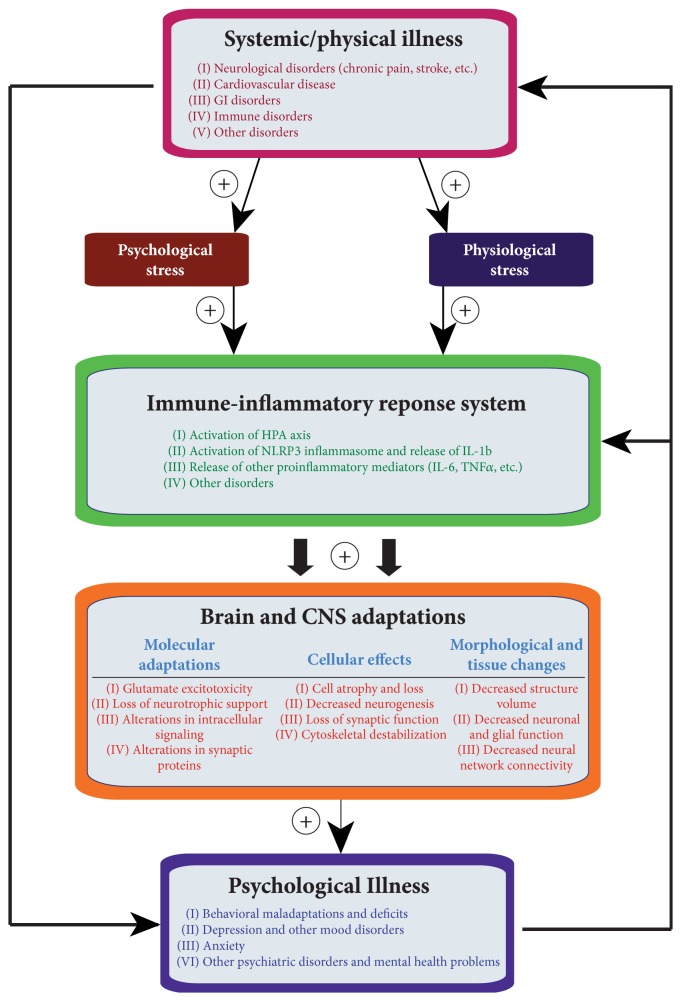
Bidirectional relationship between systemic illness and psychiatric disorders. Physical and/or psychological stress associated with systemic illness can lead to activation of immune response system resulting in increased local and systemic release of proinflammatory cytokines. Increased levels of inflammatory mediators in the CNS are potentially key contributors to the damaging cellular and morphological adaptations that underlie development of comorbid mental illness.

Clinical and basic science research has provided strong evidence that prolonged exposure to psychological stress can lead to the development of behavioral deficits and psychiatric illness, especially depression and anxiety. Stress affects the nervous system and brain as a whole; however, several limbic areas within the mood-regulating neurocircuitry, such as the hippocampus and prefrontal cortex (PFC), seem to be especially vulnerable to chronic stress [6, 7]. The negative effects of stress have been consistently observed in these brain structures, especially morphological and molecular adaptations shown to impair normal neural and glial cell function (Figure 1). For example, examinations of stressed brains in rodent models, as well as imaging studies and postmortem analysis of depressed human brains, have revealed significant atrophy (e.g., reductions in dendritic arborization and spine density), cell loss, and reduced tissue volume in the hippocampal and PFC regions [8–16]. Decreased density and number of glial cells, both astrocytes and oligodendrocytes, have also been observed in depressed and bipolar brains, within the dorsolateral PFC, orbitofrontal cortex [17, 18], and anterior cingulate cortex [10, 19]. Furthermore, stress was also shown to decrease neurogenesis (i.e., production of new neurons) in the adult hippocampus. Although the exact role of neurogenesis in the etiology and maintenance of depression is still unclear, currently available reports indicate that neurogenesis is increased by antidepressant treatments and may be required for development of antidepressant behavioral responses [20–23]. At the molecular and biochemical levels, stress initially evokes responses such as increased glucocorticoid release and altered neurotransmitter activity, followed by glutamatergic excitotoxicity, decreased neurotrophic support, and diminished synaptic function [24–26]. With chronic exposure to stress, these initial adjustments may progress into cellular destabilization and morphological changes eventually resulting in dysfunction of both individual neurons and entire neural networks encompassing both limbic and cortical brain areas [7].

### 2.2. Role of Neurotrophic Factors and Proinflammatory Cytokines

Stress-evoked brain alterations mentioned above may represent some of the key neuropathological factors underlying development of behavioral deficits and an overall depressive phenotype; however, the exact molecular adaptations and subsequent cellular remodeling mechanisms are still not well defined. During the last three decades, a number of different modulators and intracellular pathways have been linked to stress responses. A plethora of evidence suggests that adequate brain levels of neurotrophic factor signaling, which is involved in neuronal growth, differentiation, and survival, may have a critical role in both the etiology of mood disorders and treatment response and, thus, have led to formation of the neurotrophic hypothesis of depression [27–29]. For example, decreased expression levels of brain-derived neurotrophic factor (BDNF), a member of nerve growth factor family, and its preferred receptor tyrosine kinase B (TrkB) were found in the hippocampus and PFC of both depressed humans and animals exposed to stress [30–32]. In contrast, chronic antidepressant treatments were shown to increase* in vivo* BDNF levels [33, 34]. Further studies also showed that intrahippocampal administration of BDNF produces antidepressant-like behavioral effects [35], while actions of chemical antidepressants are attenuated in BDNF knockout mice [36]. Moreover, actions of BDNF in this framework also seem to be multifaceted and brain circuitry-dependent, as its increased activity within the mesolimbic dopamine reward pathway (i.e., ventral tegmental area and nucleus accumbens) results in development of depressive-like behaviors [37]. However, when administered by routes that impact multiple brain areas and different neuronal circuits (i.e., intracerebroventricular or systemic), BDNF largely produces antidepressant responses [38, 39]. In addition to BDNF, a number of recent studies using combinations of* in vivo* and* in vitro* approaches have identified several additional key neurotrophic factors that are involved in stress processing and/or antidepressant actions including fibroblast growth factor 2 (FGF2), neuritin, vascular endothelial growth factor (VEGF), insulin-like growth factor 1 (IGF-1), and VGF (nonacronymic);* for a more comprehensive review see* [7, 23].

Activated immune cells, such as macrophages, T-lymphocytes, and CNS's microglia, synthesize and secrete cytokines that function as signaling molecules implicated in regulation of cellular homeostasis and chronic inflammatory processes [40]. The idea that immune system activation may also be associated with stress and depression pathophysiology (Figure 1) is supported by clinical findings showing elevated blood levels of proinflammatory cytokines (PICs), primarily interleukin- (IL-) 1*β*, IL-6, and tumor necrosis factor alpha (TNF*α*), in depressed patients [41, 42]. Likewise, robust upregulation of genes coding for different inflammatory cytokines was found in a recent microarray analysis of depressed PFC [43]. Overall, increased activity of PICs is thought to contribute to the CNS effects of stress to induce a depressive phenotype, including symptoms such as anhedonia and sleeping abnormalities [41]. Additional evidence linking the negative effects of stress and PICs comes from recent reports suggesting that stress-induced activation of IL-1*β* leads to inhibition of neurogenesis in animal models [44–46]. Specifically, administration of IL-1*β* was shown to suppress cell proliferation within the hippocampal dentate gyrus subregion, while blockade of IL-1*β* signaling was protective against antineurogenic effects and depressive-like behaviors caused by stress [44].

### 2.3. Altered Neuronal Plasticity, Synaptic Dysfunction, and Synaptogenesis

Recent studies have revealed that stress and depression are also associated with decreased synaptic function and reduced overall neural connectivity in the PFC and hippocampus, possibly due to loss of neurotrophic signaling and glucocorticoid overactivity in these brain areas [47]. Neural processes involved in synaptic plasticity and synaptogenesis (i.e., production of new neuronal synapses) were shown to be particularly susceptible to stress and may play a critical role in antidepressant effect [48, 49]. This idea is further supported by evidence demonstrating that administration of drugs such as ketamine (a glutamate receptor antagonist) evokes robust increases in synaptogenesis resulting in a rapid antidepressant response, even in treatment-resistant depressed patients [50–52]. Moreover, these rapid actions of ketamine are contingent upon increased BDNF release and activation of mammalian target of rapamycin (mTOR) intracellular pathways, leading to downstream production of synaptic proteins involved in formation of new dendritic spines and synaptogenesis [53, 54]. Thus, enhanced synaptic activity and neural network connectivity, especially within the PFC, are thought to underlie rapid antidepressant behavioral responses and provide new directions for development of improved treatment and diagnosis of depression and other psychiatric illnesses.

## 3. Comorbidity of Stress-Related Mood Disorders and Systemic Illness

### 3.1. Neurological Disorders

#### 3.1.1. Chronic Pain

Chronic pain is commonly associated with altered mood. Numerous clinical reports indicate the high prevalence of depression and other psychiatric illnesses among patients with all types of chronic pain conditions [55–59]. It is estimated that comorbid depression can be present in 30–50% of clinical chronic pain patients [60]. Although the physiological mechanisms linking pain and mood disorders are not clear, recent human and animal studies have demonstrated that pain produces morphological, organizational, and functional changes in both cortical and subcortical brain structures [61]. Neuroimaging studies have revealed that direct pain exposure activates sites within the brain network (i.e., “pain matrix”) that are responsible for defining cognitive and emotional aspect of pain and are mostly independent of sites involved in the sensory pain processing [62–66]. This component of pain-activated neurologic signature [67] involves primarily limbic neurocircuitry, including brain areas such as the anterior cingulate cortex, insula, PFC, amygdala, and hippocampus, and is thought to also help incorporate aspects such as attention, anticipation, memory, and empathy into the formation and characterization of the overall pain experience and perception [68–71]. However, in chronic pain states, prolonged nociceptive activation of the limbic neurocircuitry may also lead to dysfunction of affective pain processing resulting in development of secondary pathologies including mental illness [72–74]. This idea is further supported by studies showing a high correlation between severity of depression and the duration and severity of pain, number of pain sites, number of pain days, frequency of breakthrough pain, and general pain-related inhibition in daily functioning [56, 59, 75–77]. Likewise, additional factors such as age, gender, level of motor dysfunction, marital status, and other socioeconomic conditions have been shown to affect the incidence and severity of pain-related depression [59, 78, 79]. Other clinical observations further suggest that the relationship between pain and mood disorders is most likely reciprocal, as depressed patients commonly experience increased pain complaints (e.g., headache and stomach pain), and the perception of endogenous pain stimuli is, in part, correlated to modality, severity, and duration of pain conditions [80, 81]. Such findings could be explained by the effects of chronic stress, via elevation of glucocorticoid levels, on the function of corticolimbic and brainstem structures that modulate endogenous pain facilitation [82, 83]. Altogether, these clinical observations suggest potential impairments of the sensory systems (i.e., ascending and descending pain-control pathways) in patients with mood disorders, as well as robust alterations in activity of stress- and mood-regulating brain areas in chronic pain patients.

Pain- and stress-evoked overactivation and dysfunction of the HPA axis may, in part, underlie the physiological and pathological events that link these disease states [84]; however, the exact CNS mechanisms are still unknown. Our previous findings have indicated that persistent pain causes cellular and molecular adjustments within the limbic brain areas similar to those evoked by prolonged stress. Specifically, we found robust decreases in neurotrophic factors signaling (e.g., BDNF) and diminished rate of neurogenesis in the hippocampus of animals exposed to 21 days of peripheral, inflammatory nociception [85–88]. Similar findings were also more recently observed in animal models of neuropathic pain, where pain-evoked decreases in hippocampal neurogenesis and synaptic deficits were linked to anxiety-like behaviors and deficits in learning and memory [89]. At the molecular level, neuropathic pain also evokes increased expression of PICs in the brain, which is thought to contribute to the development of depressive-like behaviors [90, 91]. Likewise, pain-related development of allodynia is correlated with sustained elevation of hippocampal PICs, specifically IL-1*β* and IL-6 [92]. Furthermore, these initial pain-evoked increases in IL-1*β* were shown to be dependent on ATP signaling and P2X7 receptor activation, which suggest involvement of microglial cells in supraspinal pain processing [93, 94]. This notion is supported by a recent study showing that neuropathic pain causes increases in activated microglia within the reward neurocircuitry leading to disruption of dopamine-mediated reward behavior [95]. In total, these preclinical reports indicate that neural mechanisms linking pain and mood alterations may be dependent on induction of neuroinflammation within the brain structures involved in regulation of emotional states and pain perception.

#### 3.1.2. Substance Use Disorders and Alcoholism

Large-scale epidemiological studies have also revealed a high extent of comorbidity between substance abuse and mood disorders [96–98]. There is a near-twofold increase in the lifetime prevalence of depression in individuals with a substance use disorder (SUD), and vice versa, reflecting the reciprocal nature of these disorders.

Several mechanisms have been proposed to underlie the high cooccurrence of SUD and mood disorders [99]. First, mood disorders and SUD may be risk factors for each other due to their negative pathological effects. In the case of alcoholism, which includes both alcohol abuse and dependence, excessive consumption of alcohol can cause negative emotional and social problems. Conversely, those suffering from mood disorders may seek alcohol and/or other drugs of abuse as self-medication to cope with their stress and anxiety symptoms, resulting in a higher risk of developing alcoholism and/or drug addiction. Furthermore, there may exist overlapping neural circuits that are targets of pathological manifestations of both SUD and stress. Recent neural imaging studies have revealed that both patients with substance dependence and mood disorders exhibit increased activation in certain regions of the brain's reward system during performance of emotional and reward-processing task;* for a more comprehensive review see* [100]. Finally, common genetic factors may also contribute to the cooccurrence [101, 102]. This idea is supported by a recent study showing that simultaneous treatment of clinically depressed alcoholic patients with both a selective serotonin reuptake inhibitor (SSRI) and an opioid antagonist (i.e., for alcohol dependence) produces a greater therapeutic effect [103]. Overall, such findings suggest that comorbidity of mood disorders and SUD require development of treatment strategies that address both disorders, preferably through combining psychosocial treatment with pharmacotherapy.

#### 3.1.3. Stroke

Different types of psychiatric disorders are commonly observed following stroke, including depression, anxiety, emotional incontinence, delusions, and hallucinations [104]. Poststroke depression (PSD) is the most frequent neuropsychiatric complication after stroke, affecting approximately 30–50% patients within the first year of recovery [104–106]. PSD not only was shown to impact cognitive function and mental health but also impairs rehabilitation progress and functional motor recovery in stroke patients. PSD is also associated with increased risk of suicide and increased mortality; however, it is frequently undetected and remains untreated [107–110]. The pathophysiology of PSD has been shown to be multifactorial, including aspects of neuroanatomical size, location and number of lesions, stroke subtype and severity, social difficulties, family support, and overall stroke burden [104]. Moreover, emerging evidence suggests that risk factors contributing to the vulnerability to depression involve neuroanatomical alterations (e.g., smaller amygdala), disruption of basal ganglia-prefrontal pathways, and cerebrovascular impairments, such as hypertension, atherosclerosis, and hyperlipidemia [111–114]. In addition to these factors, preexisting depression is another independent risk factor for stroke development and recurrence, indicating a reciprocal association between these disease processes [115, 116]. Overall, the association between stroke and depression is well documented; however, the etiology of PSD is highly complex and remains poorly understood.

#### 3.1.4. Alzheimer's Disease

Several meta-analyses have linked a history of clinical depression or depressive-like symptoms with Alzheimer's Disease (AD) [117–120]. Specifically, late-life depression appears to be a significant risk factor for future development of AD [121–123]. Similarly, patients with a history of depression prior to the diagnosis of AD are more likely to experience new depressive episodes throughout the course of AD [124, 125] and the corresponding depressive symptoms tend to worsen with progression of the disease [126]. Although there is significant clinical evidence supporting this comorbidity between depression and AD, very little is understood about the neural mechanisms linking the two disease states. Recent studies show that rodent models of AD do exhibit depressive-like behavioral deficits such as helplessness, indicated by prolonged immobility in the forced swim test [127]. Furthermore, at the molecular level, decreased BDNF mRNA expression and protein levels were found in the postmortem AD brains, especially within the areas linked to depression pathophysiology such as the hippocampus and frontal cortex [128–131]. These observations of depleted growth factor levels in the AD brains are similar to previous findings in depressed brains and may underlie the progressive cell death and neurodegeneration characteristic to both illnesses. Thus, overall lack of neurotrophic support in key brain areas may represent one common pathogenic mechanism linking AD and depression.

Numerous studies also report altered levels of various cytokines in the peripheral circulation of AD patients [132–134]. More importantly, general patterns of rise in the serum levels of several PICs, including IL-1*β*, TNF*α*, and IL-6, observed in AD patients [133, 135] are very similar to increases in circulating levels of proinflammatory cytokines in depressed patients [136, 137]. Interestingly, in patients with comorbid AD and depression, blood PIC levels are even more elevated [138]. Moreover, these peripheral immune studies are seemingly corroborated by genetic studies investigating the involvement of alleles associated with higher production of PICs in a sample of elderly patients with AD and depression [139]. In sum, besides alterations in growth factor activity within the brain, changes in the activation of peripheral immune system may signify another pathogenic mechanism linking clinical features of AD and various mood disorders, including depression.

### 3.2. Cardiovascular Disease (CVD)

Cardiovascular health is influenced by many factors. Traditionally, the major risk factors for cardiovascular disease are high blood pressure, high blood lipids, diabetes, and family history. However, other risk factors have recently emerged to predict cardiovascular health, especially ones that include lifestyle choices (e.g., exercise and nutrition). Moreover, emerging research has identified chronic stress as another important risk factor for development of cardiovascular disease. This idea is supported by a number of clinical studies that have established a strong link between prolonged exposures to psychological stress and increased incidences of stroke, myocardial infarction, atherosclerosis, and coronary artery disease [140–145].

The risk for myocardial infarction (MI) can also be significantly increased by previous exposures to psychological stressors, as demonstrated by the* INTERHEART* study [141]. In the Nurse's Health Study 2 respondents suffering posttraumatic stress disorder (PTSD, by the clinical definition) were shown to have a 60% enhanced risk of MI or stroke [145]. Furthermore, this study also found the presence of additional behavioral risk factors (e.g., smoking and sedentary lifestyle) among subjects at highest risk of MI. The relationship between chronic stress and behavioral risk factors has also been shown in other cohorts [146, 147]. For example, chronic stress is associated with poor food choices, leading to obesity and metabolic disorders, which are independent risk factors for CVD. A recent study by Bergmann and colleagues examined the role of chronic stress on the development of the metabolic syndrome, a constellation of disorders including obesity, high blood pressure, hyperglycemia/insulin resistance, and hyperlipidemia [148]. Interestingly, the energy homeostasis was shown to be significantly impaired in stressed animals in comparison to control animals when both were fed a high-fat diet. Stressed animals on high-fat diet also exhibited altered levels of leptin, the “satiety hormone” previously shown to have the capability to change the activity of specific brain regions, including those involved in control of sympathetic outflow and regulation of stress responses [149–151]. Moreover, presence of stress-related hyperglycemia has also been documented and implicated in increased activation of proinflammatory signaling and promotion of oxidant stress [152, 153].

Recent human and animal studies have further demonstrated that stress can also lead to the development of vascular dysfunction resulting in a predisposition to CVD [154–157]. In addition, both acute and chronic stressors have been shown to impair vascular reactivity. Although the exact mechanism and effect seem to be dependent on the type of the stressor and/or the model, there is evidence to suggest that reduced vascular dilation and enhanced constriction may play an important role [156, 157]. In humans, acute psychological stress reduces the magnitude of flow-mediated dilation of the brachial artery, a measure of the reactivity of the peripheral vasculature that is thought to be the result of altered nitric oxide handling [154]. Furthermore, in the Takotsubo syndrome, an acute coronary condition usually resulting from an exposure to a major emotional or physical stressor, a reaction is triggered that mimics coronary artery disease, which is suggested to be a result of increased sympathetic activity leading to impaired coronary vascular reactivity [158]. Animal studies have also shown changes in coronary hemodynamics as a result of psychological stress. For example, studies in dogs have demonstrated that psychological stress can reduce coronary blood flow, resulting in electrocardiographic changes resembling an acute MI, and an enhanced risk for arrhythmias, possibly through alterations in the effective refractory period [159, 160]. Similarly to chronic stress, a number of recent studies have linked depression to peripheral arterial disease [161], coronary artery disease [162], MI/stroke [163, 164], heart failure [165–167], and hypertension [168, 169], suggesting that depression, perhaps as a result of sustained stressors, can also have a major impact on cardiovascular health.

In both clinical and preclinical studies, chronic stress has been shown to increase proinflammatory signaling cascades and reactive oxygen species, enhance vasoactive agents (e.g., endothelin-1 and angiotensin II), and activate both the HPA axis and sympathetic nervous system activity, while reducing the activity of vasodilators (i.e., nitric oxide) and abrogating the activity of the parasympathetic nervous system. Sympathetic activity is also increased with stress, mainly due to enhanced neuronal discharge from the paraventricular nucleus of the hypothalamus and the rostral ventrolateral medulla, leading to overactivation of sympathetic preganglionic neurons [170, 171]. Additionally, neurohumoral factors including angiotensin II are also increased in chronic stress [172, 173]. Angiotensin II is linked to cardiovascular disease through direct vasoconstriction, altered salt and water excretion from the kidney, and cellular remodeling. Moreover, angiotensin II can perpetuate the proinflammatory state through activation of nuclear factor- (NF-) *κ*B, among other transcription factors [174–177].

### 3.3. Gastrointestinal Disorders

Gastrointestinal (GI) disorders are often associated with stressful symptoms including chronic abdominal pain, severe diarrhea, and changed bowel habits. Some of the major functional/structural GI disorders, such as the inflammatory bowel disease (IBD), are characterized by chronic inflammation of the digestive tract; however, there are no identifiable anatomic or biochemical abnormalities that can be attributed to the symptoms. In addition to the symptoms of physical illness, patients with GI disorders commonly exhibit mental health problems and are often diagnosed with psychiatric illness. Depression and mood disorders are among the most common psychiatric comorbidities with a much higher prevalence than the general population [178]. The Manitoba IBD cohort study reported a lifetime depression rate of 27% in IBD patients compared to 12% in healthy controls [179]. Similarly to other systemic illnesses, the relationship between mood and GI disorders appears to be bidirectional. Patients with depression have been found to have a significantly higher frequency of IBS and at greater risk for subsequent development of GI symptoms [180].

Potential mechanisms linking GI and mood disorders include, but are not limited to, alterations in brain regions involved in emotion regulation, gut-brain axis, HPA axis, and proinflammatory signaling. There is increasing evidence suggesting a bidirectional communication between the gut-residing microbiota/microbiome and the brain [181, 182]. This interaction has been demonstrated in animal models where perception of stress by the brain was shown to reduce the diversity of the microbiota in the gut [183] and, in turn, the microbiome also influences stress responses and depressive behaviors through modulating brain activity [184]. Recent clinical studies further support this relationship as chronically depressed patients were shown to have increased plasma levels of immunoglobulins (Ig), especially IgA and IgM, indicating enhanced immune responses against lipopolysaccharides (LPS) of different commensal Gram-negative bacteria [185, 186]. This type of systemic activation of IgA/M-mediated inflammatory response system (IRS) suggests that greater permeability of the gut wall (i.e., “leaky gut”) and enhanced bacterial translocation may signify additional phenomena that can play important roles in development of both sickness behavior symptoms and pathophysiology of chronic depression [187].

At the molecular level, multiple genetic factors may contribute to the comorbid occurrence of mood and GI disorders, including polymorphisms of BDNF and serotonin transporter genes [188, 189]. It is also worth noting that in female IBS patients enhanced tryptophan degradation has been detected, which results in depletion of tryptophan, the precursor for serotonin synthesis [190]. Thus, it is plausible that hypofunction of serotonin links IBS with depression. Moreover, symptoms associated with physical aspects of GI disorders, such as chronic pain and inflammation, may also serve as risk factors for development of depression. Conversely, mood disorders in turn can adversely affect the course of GI disorders as well as the treatment outcomes. More effective treatments, aimed at breaking this vicious circle, are needed with the intention to mitigate the comorbid mood disorders simultaneously [191]. These treatments, both pharmacotherapy (e.g., antidepressants) and psychotherapy, should demonstrate effectiveness in the context of depression cooccurring with GI symptoms.

### 3.4. Obesity and Metabolic Disorders

In addition to the above-mentioned disorders, systemic activation of innate immune processes has been linked to a range of different factors that represent potential risks for comorbid depression including psychosocial stressors, daily practices, and lifestyle choices (i.e., lack of exercise and physical activity, sleep deprivation, smoking, poor diet, etc.) as well as pathological states such as obesity and metabolic disorders;* for a more comprehensive review see* [187, 192]. Obesity is usually linked to poor dietary habits and currently represents a significant public health and medical concern in terms of both prevalence and related secondary physical illnesses, respectively [193–195]. Obesity is also associated with cognitive deficits and number of comorbid mood disorders. More specifically, obesity and depression seem to be correlated in a bidirectional manner, as clinical studies have shown that obesity predisposes to the development of clinical depression, while depression can significantly increase the risk of developing obesity [187, 196, 197]. Moreover, obesity is thought to induce a state of systemic low-grade inflammation characterized by increased production of PICs that promote development of other disorders including diabetes, cardiovascular dysfunction, and psychiatric illnesses [198]. Indeed, adipose tissue contributes to development of systemic inflammation through the release of adipokines such as proinflammatory leptin and predominantly anti-inflammatory adiponectin [199]. Thus, controlling the release and activity of these adipokines is essential for maintenance of energy balance and overall homeostasis. Moreover, clinical studies have shown that obesity is associated with a reduction in adiponectin, which potentially underlies the development of type 2 diabetes (T2D), hypertension, and atherosclerosis [200]. The exact actions of these circulating metabolic regulators on CNS and brain function are still being investigated; however, several recent studies have linked alterations in central metabolic mechanisms with systemic dysregulation [201]. Additional factors including dysbiosis and excessive nutrient intake as well as different “disease clusters,” such as metabolic syndrome (MetS) and polycystic ovarian syndrome (PCOS), have also been associated with proinflammatory processes leading to obesity, metabolic dysregulation, and impaired innate immunity [202–206]; however, further research is necessary to determine how these processes and pathological states influence chronic psychological dysfunction and psychiatric illnesses.

## 4. Role of Inflammatory Processes in Linking Stress and Systemic Illness

**Figure 2:**
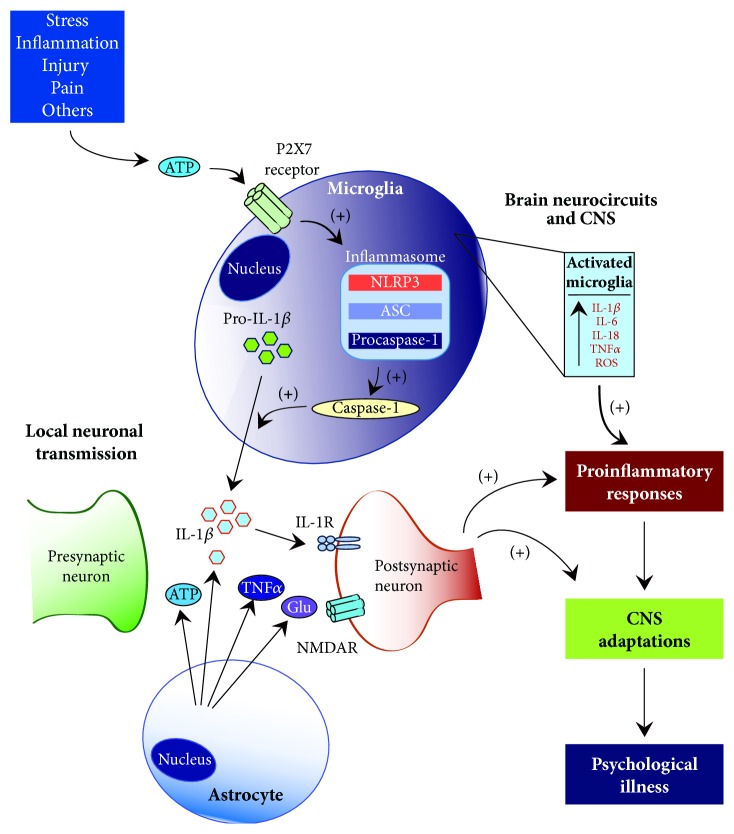
Role of microglia and inflammasome complex in development of inflammatory responses. Systemic disorders and pathological states can induce activation of microglial cells leading to a local release of proinflammatory cytokines (PICs). This process includes stimulation of microglial P2X7 receptors and downstream activation of the NLRP3 inflammasome. Consequent systemic increases in circulating PICs may signify one of the fundamental mechanisms responsible for initiation of molecular and functional alteration within the CNS that underlie development of mental illness. Interleukin (IL), tumor necrosis factor alpha (TNF*α*), reactive oxygen species (ROS), NOD-like receptor family, pyrin domain containing 3 (NLRP3), apoptosis-associated speck-like protein containing a CARD (ASC),* N*-methyl-D-aspartate receptor (NMDAR), and purinergic type 2X7 (P2X7).

Based on the clinical and preclinical evidence discussed so far, the elucidation of the precise mechanisms underlying how psychological and physical stressors can influence the CNS and peripheral organ systems as well as the development of systemic diseases remains unsolved. A recently proposed immune-inflammation based hypothesis of depression and related comorbid systemic illnesses may help answer this question [5]. This hypothesis suggests that an inflammasome protein complex and related inflammatory reactions are the main driving force underlying the reciprocal/bidirectional relationship between the psychological stress-related psychiatric illness and comorbid systemic disease (Figure 2).

The idea that psychological and physiological stressors can stimulate inflammatory cytokine activity, leading to neuronal dysfunction and ensuing development of psychiatric illness such as MDD, has been promoted for over 30 years [207, 208]. Although exact physiological and molecular pathways are not fully understood, recent findings have demonstrated/indicated bidirectional/reciprocal communication between neuroendocrine and immune systems [209]. As previously mentioned, several meta-analysis studies have provided strong evidence that depression is accompanied by alterations in immune system function and activation of inflammatory response system (IRS) as increased levels of PICs (i.e., IL-1*β*, IL-6, and TNF*α*) were found in the blood of depressed patients [210, 211]. In contrast, serum levels of these cytokines are normalized by treatments with tricyclic antidepressants (TCAs) and SSRIs [212]. Furthermore, increased levels of PICs (i.e., IL-1*β* and TNF*α*) were also found in brain tissue after exposure to psychological stressors, an observation consistent with their role as potent activators of the HPA axis, which is commonly dysregulated in mood disorders [213, 214]. For example, approximately two-thirds of patients suffering from depression exhibit elevated cortisol levels [215]. At the cellular level, cytokines are involved in release of different stress hormones implicated in HPA axis activity, primarily the CRH and ACTH [216, 217]. Moreover, enhanced activation of these cytokines has been linked with sickness behaviors in animal models and mood alterations, such as dysphoria and anxiety, that resemble behavioral symptoms of depression in humans [209]. Commonly, the release of these proinflammatory cytokines transpires as a result of activation of the innate immune system defense mechanisms in response to the presence of potentially survival-threatening pathogenic molecules and/or an array of danger signals [5]. In line with this idea, preclinical studies have identified IL-1*β* as a critical mediator of stress-induced behaviors. Several lines of empirical evidence support this notion: (1) stress was shown to increase hippocampal IL-1*β* levels, (2) IL-1*β* robustly suppresses hippocampal cell proliferation, and (3) genetic deletion or pharmacological blockade of IL-1*β* receptor completely protects against damaging effects of stress and development of depressive-like behaviors [44]. In addition, these IL-1*β*-related prodepressive effects are thought to occur through activation of the NF-k*β* signaling pathway [218].

Recent mechanistic studies have identified toll-like receptors (TLRs) as key contributors to the recognition and initiation of an immune response in the presence of pathogenic molecules leading to increased gene transcription and release of IL-1*β*, IL-6, and TNF*α* [219, 220]. Moreover, the induction and final release of mature IL-1*β* protein also entail cleavage from inactive pro-IL-1*β* form. This process was shown to be dependent on stimulation of the ATP purinergic type 2X7 (P2X7) receptors and downstream activation of the NLRP3 inflammasome complex that includes caspase-1, an enzyme responsible for cleavage of pro-IL-1*β* (Figure 2) [221–224]. In the CNS, the NLRP3 inflammasome is constitutively expressed in microglial cells which are present throughout the brain, especially in the hippocampus, a mood-regulating area susceptible to chronic stress [5]. Finally, the recently proposed idea that activation of NLRP3 inflammasome is the central mechanism linking systemic illness and comorbid mood disorders is further supported by observations that a number of different neurological, cardiovascular, metabolic, and inflammatory diseases have been associated with NLRP3 and elevated IL-1*β* release;* for a more comprehensive review see* [5]. Altogether, these findings suggest that inflammatory cytokines and NLRP3 inflammasome may act as key neuromodulators in neurochemical and physiological alterations that underlie development and maintenance of behavioral deficits associated with both mood disorders and systemic illnesses.

## 5. Conclusions

Significant amounts of evidence from human and animal research have demonstrated that psychological and/or physical stress is a powerful driving force behind both the pathophysiology of and comorbidity between psychiatric and systemic disorders. Stress-related detrimental modifications in structure, organization, and function of CNS may, in part, explain the coincidence and comorbidity between these pathological states. This concept is further supported by clinical observations showing that mood disorders, such as depression, are some of the most prevalent and debilitating psychiatric illnesses associated with other systemic disorders. Recent findings point towards the immunoinflammatory system as a key endogenous response mechanism involved in this elusive, bidirectional interaction between stress and illness; however, further studies are needed to reveal the exact pathophysiological mechanism(s) involved. Nonetheless, the question remains whether the mental health (e.g., behavioral/mood deficits) should be addressed as part of the standard treatment of systemic disease. Future studies may link different areas of biomedical sciences and open new research avenues that may ultimately identify novel clinical strategies for improved management of systemic illness by also improving the mental health aspects of these disorders.
